# Renal Cell Carcinoma and Visceral Adipose Index: a new risk parameter

**DOI:** 10.1590/S1677-5538.IBJU.2015.0396

**Published:** 2016

**Authors:** Alper Otunctemur, Murat Dursun, Kutan Ozer, Ozan Horsanali, Emin Ozbek

**Affiliations:** 1 Department of Urology, Okmeydani, Training and Research Hospital, Istanbul, Turkey;; 2 Department of Urology, Ataturk Training and Research Hospital, Izmir, Turkey

**Keywords:** Carcinoma, Renal Cell, Visceral Pain, Hypertension, Body Mass Index

## Abstract

**Purpose:**

The aim of this study was to evaluate the relationship between tumor size and grade with visceral adipose index (VAI) levels in patients with renal cell carcinoma.

**Materials and methods:**

We retrospectively reviewed the records of 310 consecutive patients with RCC who underwent radical nephrectomy at our institution between January 2007 and May 2014. VAI was calculates for males and females seperately as this formula like previous study. The relationship between tumor size and nuclear grade with VAI levels were evaluated statisticaly. Analyses were completed using Chi-square tests and Logistic regression analysis.

**Results:**

Among the 310 total patients analyzed in our study, there were 176 males (56.8%) and 134 females (43.2%). VAI levels were statistically higher in men and women with high tumor size (p<0.001). VAI levels were statistically higher in men and women with high fuhrman grade (p<0.001).

**Conclusions:**

The components of VAI may have effect on tumor carcinogenesis in similar pathways. In our study patients with high VAI levels were found to have statistically significant higher nuclear grade and tumor size. VAI can be a useful index for the evaluation and calculation of renal cell cancer aggressiviness. Further studies with more patients are needed to confirm our study.

## INTRODUCTION

Renal cell carcinoma (RCC) accounts for approximately 3% of all adult malignancies, representing the seventh most common cancer in men and the ninth most common cancer in women. Based on current guidelines, surgery remains the only curative treatment option in patients with localized renal cell carcinoma (RCC) (1-3). It is a disease typically presenting in elderly patients with the mean age at diagnosis being around 60 years (4). RCC involving the renal parenchyma accounts for the majority of cases. The predominant subtype of RCC is clear cell type that represents 80% of RCC and is derived from the tubular epithelium. Other types of RCC are papillary (15%), chromophobe (5%), and collecting duct (5, 6).

Several well-established life-style risk factors, such as BMI, hypertension, and smoking, have been identified as potentially predisposing to renal cell carcinoma development (7). Previous studies have reported that diabetes type 2 among women and high BMI and blood pressure among men are independent risk factors for RCC, however, those studies had no data on blood lipids (8, 9). Body fat is known to have biological functions, such as the ability to alter lipid metabolism, modulate numerous adipokines and contribute to chronic inflammation, among which visceral fat is more metabolically active than peripheral s.c. fat (10). Body fat misdistribution, e.g. visceral obesity, is strongly associated with increased risk of insulin resistance, metabolic syndrome and cardiovascular disease than BMI alone (11). In a previous study, the authors showed that the identification of a routinely applicable indicator for the evaluation of visceral adipose function, with higher sensitivity and specificity than classical parameters [such as waist circumference (WC), BMI, and lipids], could be useful for cardiometabolic risk assessment (12). They calculate a model of adipose distribution (MOAD). To correct MOAD for fat function, TG (mmol/l) and HDL (mmoL/L) levels were introduced in the formula. They used the visceral adipose index (VAI) as this formula for cardiometabolic risk assessment.

In our previous study, we reported that patients with metabolic syndrome were found to have statistically significant higher nuclear grade and tumor size (13). Studies show that individuals with BMI 30kg/m^2^ or less, visceral obesity has been shown to be a better marker of health issues related to being overweight, eg cardiovascular disease and metabolic syndrome, than BMI itself. In this context, we thought that we could use the VAI for the assessment of RCC aggressiviness. So, the aim of this study was to evaluate the relationship between tumor size and grade with VAI levels in patients with renal cell carcinoma.

## MATERIAL AND METHODS

We retrospectively reviewed the records of 310 consecutive patients with RCC who underwent radical nephrectomy at our institution between January 2007 and May 2014. We analyzed the following clinicopathologic variables: age, gender, the presence of hypertension, diabetes, body mass index (BMI), waist circumference, tumor size, histologic subtype, Fuhrman nuclear grade, HDL and trigliceride levels. Plasma fasting glucose, high-density lipoprotein (HDL) cholesterol levels and triglycerides were measured using enzymatic methods with an autoanalyzer. Pathologic staging was performed using the 7th edition of the American Joint Committee on Cancer (AJCC). Histologic subtype was determined according to the 1997 World Health Organization Heidelberg classification and tumor nuclear grading was performed according to the Fuhrman nuclear grading system.

VAI was calculated for males and females separatly according to this formula proposed in a previous study (12):

VAI: WC/ [39.68+(1.88 x BMI)] x TG/1.03 × 1.31/HDL (for male).
VAI: WC/ [36.58+(1.89 x BMI)] x TG/0.81 × 1.52/HDL (for female).
WC: waist circumference, BMI: body mass index,
TG: tryglyceride, HDL: high density lipoprotein.

The relationship between tumor size and nuclear grade with VAI levels were evaluated statisticaly. Local ethics committee approval had been obtained before the commence of the study. Analyses were completed using Chi-square tests and Logistic regression analysis. All statistical tests were two-tailed, and statistical significance was defined as P<0.05. All analysis were conducted using SPSS version 15.0 (SPSS Inc., Chicago, Illinois, USA).

## RESULTS

Among the 310 total patients analyzed in our study, there were 176 males (56.8%) and 134 females (43.2%). We divided the patients in two groups (tumor size ≥7cm and <7cm). BMI were statistically higher in patients with tumor size ≥7cm than <7cm (p<0.001). Comparison of the men and women with tumor size, the patients with high tumor size had higher TG levels, higher WC and lower HDL-C levels. The difference was statistically significant for all values. Also, we compared the VAI levels between two groups. In men, mean VAI level was 5.26±2.44 in group-1 and 3.58±1.84 in group-2, respectively. In women, mean VAI level was 5.21±2.61 in group-1 and 3.49±1.78 in group-2. VAI levels were statistically higher in men and women with high tumor size (p<0.001). Characteristics of all patients are shown in Table-1.

**Table 1:** Characteristics of the patients and comprasion to tumor size.

	Men with tumor size >7 cm	Men with tumor size < 7 cm	Women with tumor size >7 cm	Women with tumor size < 7 cm	p value
**Age(mean-range)**	66.5±12.34	62.8±8.94	68.8±14.21	60.8±9.56	0.52
**BMI(mean±sd)**	28.36±2.87	26.84±2.21	29.07±3.05	27.39±3.41	<0.001
**Weight(kg, mean±sd)**	78.64±14.64	77.42±16.55	74.41±11.48	72.15±13.26	0.41
**Height(meter, mean±sd)**	1.68±0.42	1.69±0.56	1.61±0.84	1.63±0.74	0.95
**WC (cm, mean±sd)**	93.77±8.27	91.03±8.51	88.54±7.16	85.16±8.82	0.004
**TG(mg/dL)(mean±sd)**	165.64±71.84	129.58±72.21	174.46±63.62	131.44±78.64	<0.001
**HDL-C(mg/dL)(mean±sd)**	41.83±7.36	47.54±4.28	38.72±7.94	45.41 ±5.36	<0.001
**VAI(mean±sd)**	5.26±2.44	3.58±1.84	5.21±2.61	3.49±1.78	<0.001

Also, we compared the VAI levels with Fuhrman grade. We divided the patients into two groups for Fuhrman grade. Fuhrman grade1 and 2 was group-1, Fuhrman grade 3 and 4 was group-2. BMI were statistically higher in patients with higher Fuhrman grade (p<0.001). Comparison of the men and women with Fuhrman grade, the patients with high Fuhrman grade had higher TG levels, higher WC and lower HDL-C levels. The difference was statistically significant for all values. Also, we compared the VAI levels between two groups. In men, mean VAI level was 5.14±2.32 in group-1 and 3.34±1.56 in group-2, respectively. In women, mean VAI level was 5.16±2.08 in group-1 and 3.09±1.84 in group-2. VAI levels were statistically higher in men and women with high Fuhrman grade (p<0.001) (Table-2). On the other hand, binary logistic regression models showed that VAI is an independently risk factor for renal cell cancer aggressiveness with higher Fuhrman grade and higher tumor size.

**Table 2:** Comparison VAI levels and Fuhrman Grade.

	Men with Fuhrman Grade 1-2	Men with Fuhrman Grade 3-4	Women with Fuhrman Grade 1-2	Women with Fuhrman Grade 3-4	p value
**VAI(mean±sd)**	3.34±1.56	5.14±2.32	3.09±1.84	5.16±2.08	<0.001

## DISCUSSION

In our study we investigated the correlation between tumor size and grade with visceral adipose index in renal cell carcinoma. Visceral abdominal obesity may have impact on tumor biogenesis since many tumoregenic factors are released from adipose tissue. Furthermore, obesity causes the changes in lipid regulation and results in insulin resistance that may foster cancer development (14). Epidemiological studies performed to date have consistently shown an increased relative risk of RCC with increases in BMI (15). Especially, increased visceral fat was found to be associated with clear-cell RCC. This study showed that, visceral fat area could constitute a primary explanation for the link between obesity and clear cell RCC (16). Conversely, Parker et al. found that patients with an increased BMI were more likely to present with a less aggressive form of RCC (17).

Recent advances have remarkably improved our understanding of the complex role of adipose tissue in carcinogenesis. Adipose tissue can secrete numerous molecules into the bloodstream, which may contribute to a protumorigenic environment. Mortality rates from RCC increase with increasing body mass in a prospective study conducted by the American Cancer Society (18). Reasons for these possibly worsened outcomes remain unclear, but may involve the production by adipose tissue of adipokines that may promote cancer growth, and dysregulated angiogenesis (19-21). Indeed, adipose tissue should be considered as an endocrine and paracrine organ that releases cytokine-like polypeptides responsible for widespread biological effects (22). In particular, adipocytes produce insulin-like growth factors, which are known to have cancer-promoting effects on renal cells and multiple angiogenic factors including VEGF and leptin. Leptin exerts direct angiogenic effects (21, 22) and upregulates VEGF mRNA expression. Increased leptin levels have been associated with RCC invasion and progression (23).

WC is a major clinical parameter used for the indirect evaluation of increased visceral fat (23). Nevertheless, WC alone does not help in distinguishing between subcutaneous and visceral fat mass. Because of that, in the previous study authors identified an index that could be used as a surrogate marker of “adipose tissue dysfunction.” VAI was significantly correlated with all metabolic syndrome factors and cardio-and cerebrovascular events (12). The previous study showed that, interestingly, VAI was independently associated with cardiovascular events. These findings might be explained by the fact that VAI includes both physical and metabolic parameters, perhaps indirectly reflecting other nonclassical risk factors, such as altered production of adipocytokines, increased lipolysis and plasma free fatty acids, which are not signified by BMI, WC, TG and HDL separately. Therefore, VAI might be a valuable index of both fat distribution and function. So, in our study we evaluated the relationship between visceral obesity and renal cell cancer aggresiveness, which was previously described.

In a previous study visceral obesity as defined by VAT% was found to be an independent prognostic factor for higher Fuhrman grade in patients with cT1a RCC. In this cohort of 186 patients with cT1a RCC we found that VAT% was an independent prognostic factor for high grade disease after adjusting for clinical characteristics and anatomical features of the tumor (24). Also, a recent study with large series showed that relative visceral obesity as assessed by VAT% was associated with clinicopathological characteristics of localized RCC. A higher VAT% at diagnosis was associated with older age at diagnosis, higher prevelance of diabetes and higher prevalance of former or current smoking status. So, a U-shaped association between VAT% quartiles and the risk of disease recurrence was observed for all patients (25). Like these studies, as an index for evaluation visceral obesity, we found VAI levels were statistically higher in men and women with high Fuhrman grade (p<0.001).

On the other hand, a recent study showed that radiologic measurement of VAT is an independent prognostic factor for Asian patients treated with targeted therapy for advanced renal cell carcinoma. In this study, interestingly the patients with low VAT had double the death rate. The explanation revealed that visceral adipose tissue may be an indicator of nutritional status in patients with advanced RCC (26).

In conclusion, the components of VAI may have effect on tumor carcinogenesis in similar pathways. In our study patients with high VAI levels were found to have statistically significant higher nuclear grade and tumor size. So, the simplicity of WC and BMI measurement and TG and HDL assessment make VAI an easily applicable index for the evaluation of visceral fat dysfunction. VAI can be a useful index for the evaluation and calculation of renal cell cancer aggressiviness. Further studies with more patients are needed to confirm our study.
